# Supplementary material for: miRNA-378 Is Downregulated by XBP1 and Inhibits Growth and Migration of Luminal Breast Cancer Cells
Source: Int J Mol Sci. 2023 Dec 22;25(1):186. doi: 10.3390/ijms25010186 (PMC10778669; doi:10.3390/ijms25010186)
Supplement: Supplementary file 1 [file ijms-25-00186-s001.zip › supplementary figures miR-378 revised sg_va.pptx]

## Slide 1
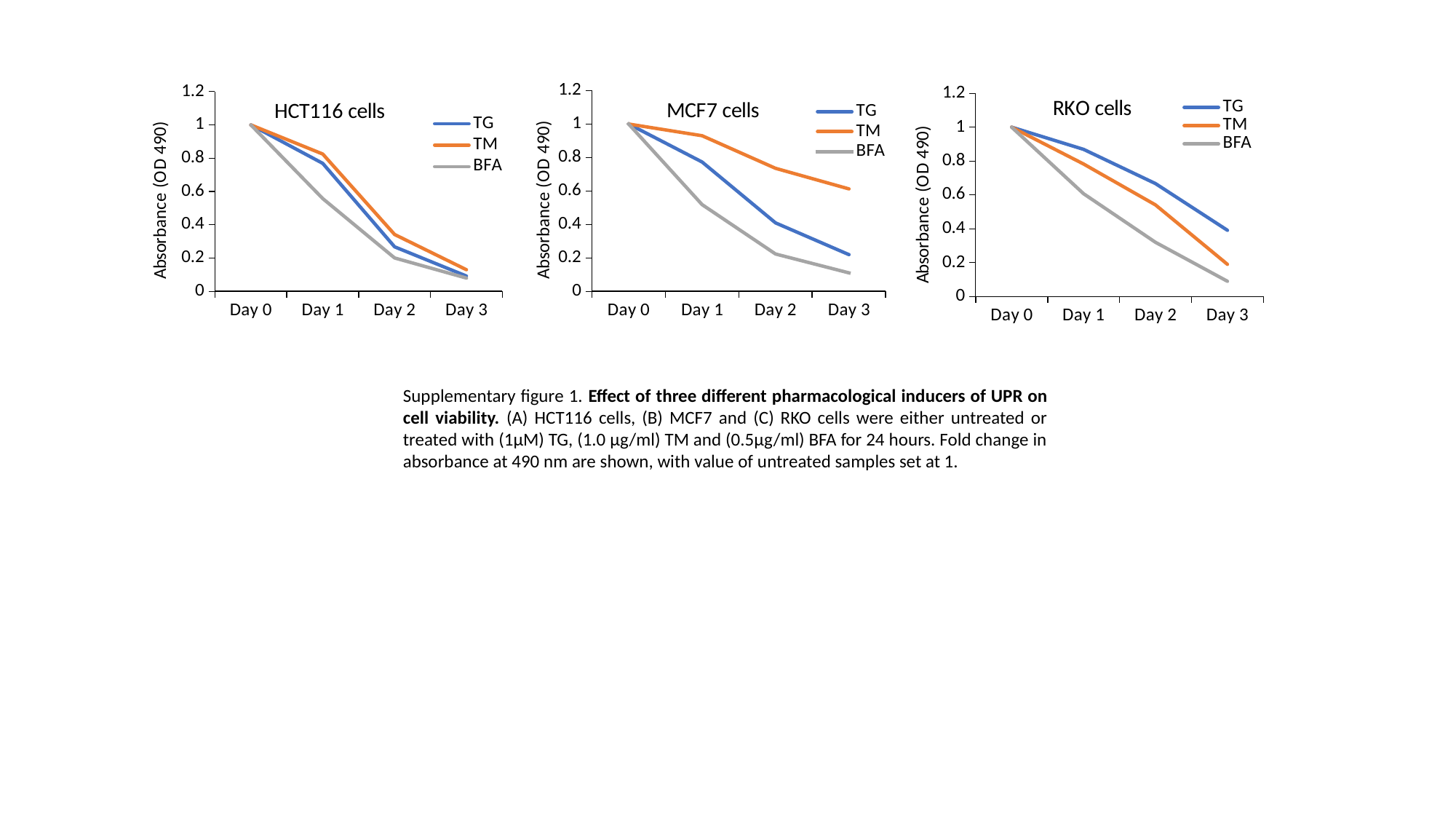

### Chart: MCF7 cells
| Category | TG | TM | BFA |
|---|---|---|---|
| Day 0 | 1.0 | 1.0 | 1.0 |
| Day 1 | 0.773 | 0.93 | 0.518 |
| Day 2 | 0.409 | 0.735 | 0.223 |
| Day 3 | 0.219 | 0.612 | 0.11 |
### Chart: RKO cells
| Category | TG | TM | BFA |
|---|---|---|---|
| Day 0 | 1.0 | 1.0 | 1.0 |
| Day 1 | 0.869 | 0.7825 | 0.607 |
| Day 2 | 0.667 | 0.541 | 0.32 |
| Day 3 | 0.391 | 0.19 | 0.09 |
### Chart: HCT116 cells
| Category | TG | TM | BFA |
|---|---|---|---|
| Day 0 | 1.0 | 1.0 | 1.0 |
| Day 1 | 0.769 | 0.825 | 0.557 |
| Day 2 | 0.267 | 0.341 | 0.2 |
| Day 3 | 0.091 | 0.129 | 0.079 |Supplementary figure 1. Effect of three different pharmacological inducers of UPR on cell viability. (A) HCT116 cells, (B) MCF7 and (C) RKO cells were either untreated or treated with (1µM) TG, (1.0 μg/ml) TM and (0.5µg/ml) BFA for 24 hours. Fold change in absorbance at 490 nm are shown, with value of untreated samples set at 1.

## Slide 2
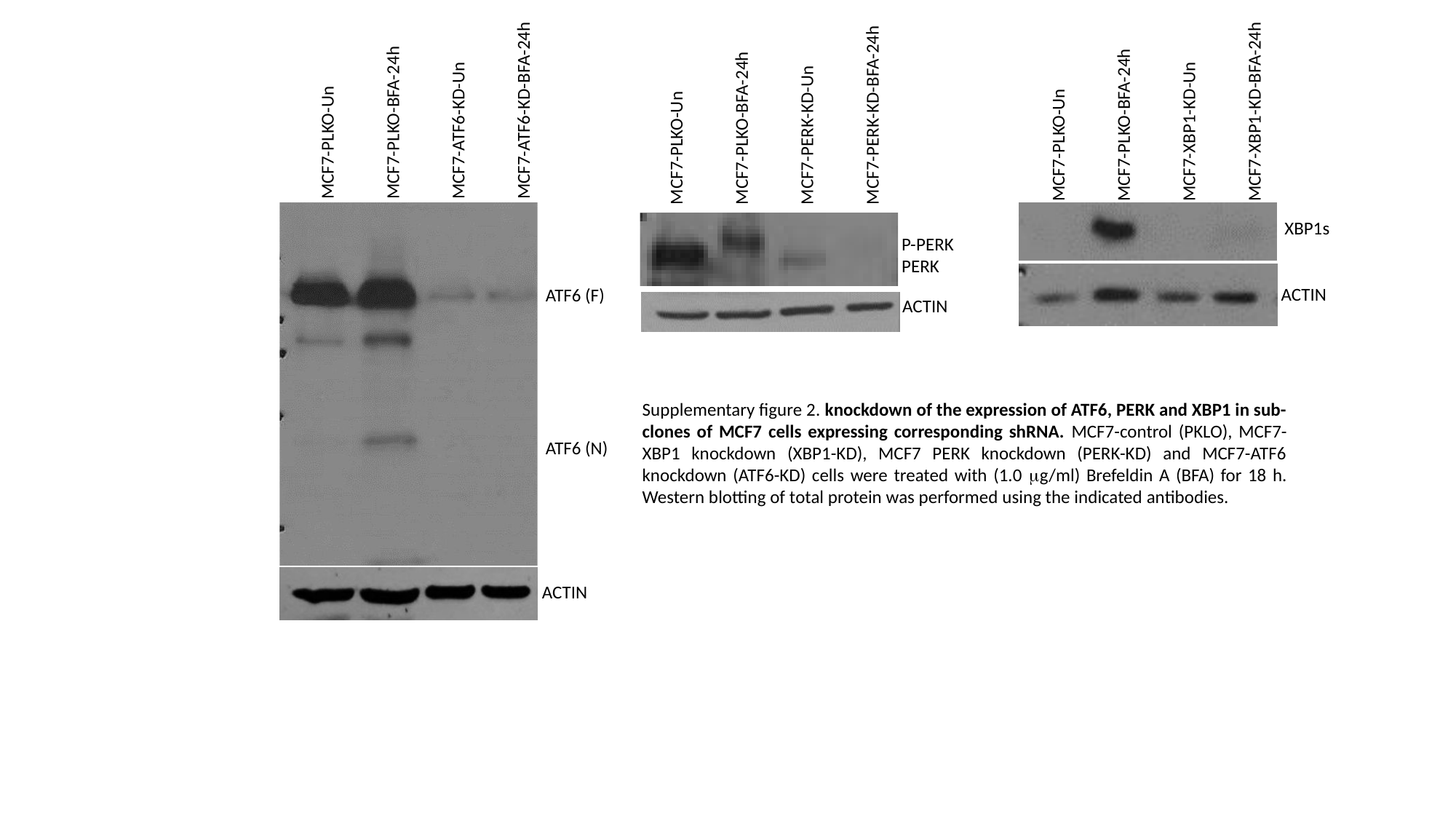

MCF7-PLKO-Un
MCF7-PLKO-BFA-24h
MCF7-XBP1-KD-Un
MCF7-XBP1-KD-BFA-24h
XBP1s
ACTIN
MCF7-PLKO-Un
MCF7-PLKO-BFA-24h
MCF7-ATF6-KD-Un
MCF7-ATF6-KD-BFA-24h
ATF6 (F)
ATF6 (N)
ACTIN
MCF7-PLKO-Un
MCF7-PLKO-BFA-24h
MCF7-PERK-KD-Un
MCF7-PERK-KD-BFA-24h
P-PERK
PERK
ACTIN
Supplementary figure 2. knockdown of the expression of ATF6, PERK and XBP1 in sub-clones of MCF7 cells expressing corresponding shRNA. MCF7-control (PKLO), MCF7-XBP1 knockdown (XBP1-KD), MCF7 PERK knockdown (PERK-KD) and MCF7-ATF6 knockdown (ATF6-KD) cells were treated with (1.0 μg/ml) Brefeldin A (BFA) for 18 h. Western blotting of total protein was performed using the indicated antibodies.

## Slide 3
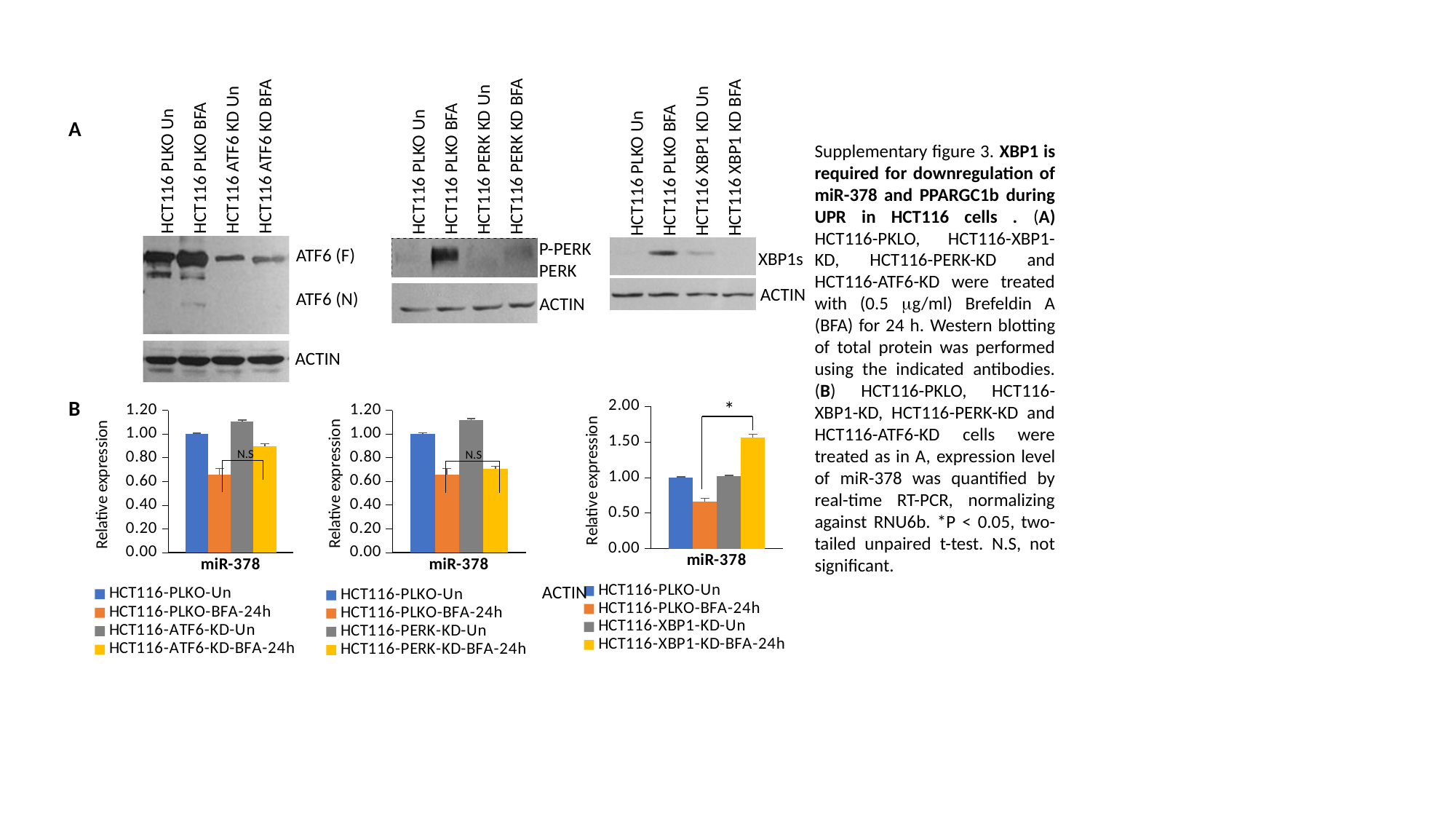

HCT116 PLKO Un
HCT116 PLKO BFA
HCT116 ATF6 KD Un
HCT116 ATF6 KD BFA
HCT116 PLKO Un
HCT116 PLKO BFA
HCT116 PERK KD Un
HCT116 PERK KD BFA
HCT116 PLKO Un
HCT116 PLKO BFA
HCT116 XBP1 KD Un
HCT116 XBP1 KD BFA
ATF6 (F)
ATF6 (N)
ACTIN
ACTIN
A
B
Supplementary figure 3. XBP1 is required for downregulation of miR-378 and PPARGC1b during UPR in HCT116 cells . (A) HCT116-PKLO, HCT116-XBP1-KD, HCT116-PERK-KD and HCT116-ATF6-KD were treated with (0.5 μg/ml) Brefeldin A (BFA) for 24 h. Western blotting of total protein was performed using the indicated antibodies. (B) HCT116-PKLO, HCT116-XBP1-KD, HCT116-PERK-KD and HCT116-ATF6-KD cells were treated as in A, expression level of miR-378 was quantified by real-time RT-PCR, normalizing against RNU6b. *P < 0.05, two-tailed unpaired t-test. N.S, not significant.
P-PERK
PERK
XBP1s
ACTIN
*
### Chart
| Category | HCT116-PLKO-Un | HCT116-PLKO-BFA-24h | HCT116-XBP1-KD-Un | HCT116-XBP1-KD-BFA-24h |
|---|---|---|---|---|
| miR-378 | 1.0004526522049804 | 0.66 | 1.02 | 1.56 |
### Chart
| Category | HCT116-PLKO-Un | HCT116-PLKO-BFA-24h | HCT116-PERK-KD-Un | HCT116-PERK-KD-BFA-24h |
|---|---|---|---|---|
| miR-378 | 1.0004526522049804 | 0.66 | 1.12 | 0.71 |
### Chart
| Category | HCT116-PLKO-Un | HCT116-PLKO-BFA-24h | HCT116-ATF6-KD-Un | HCT116-ATF6-KD-BFA-24h |
|---|---|---|---|---|
| miR-378 | 1.0004526522049804 | 0.66 | 1.11 | 0.9 |N.S
N.S
ACTIN

## Slide 4
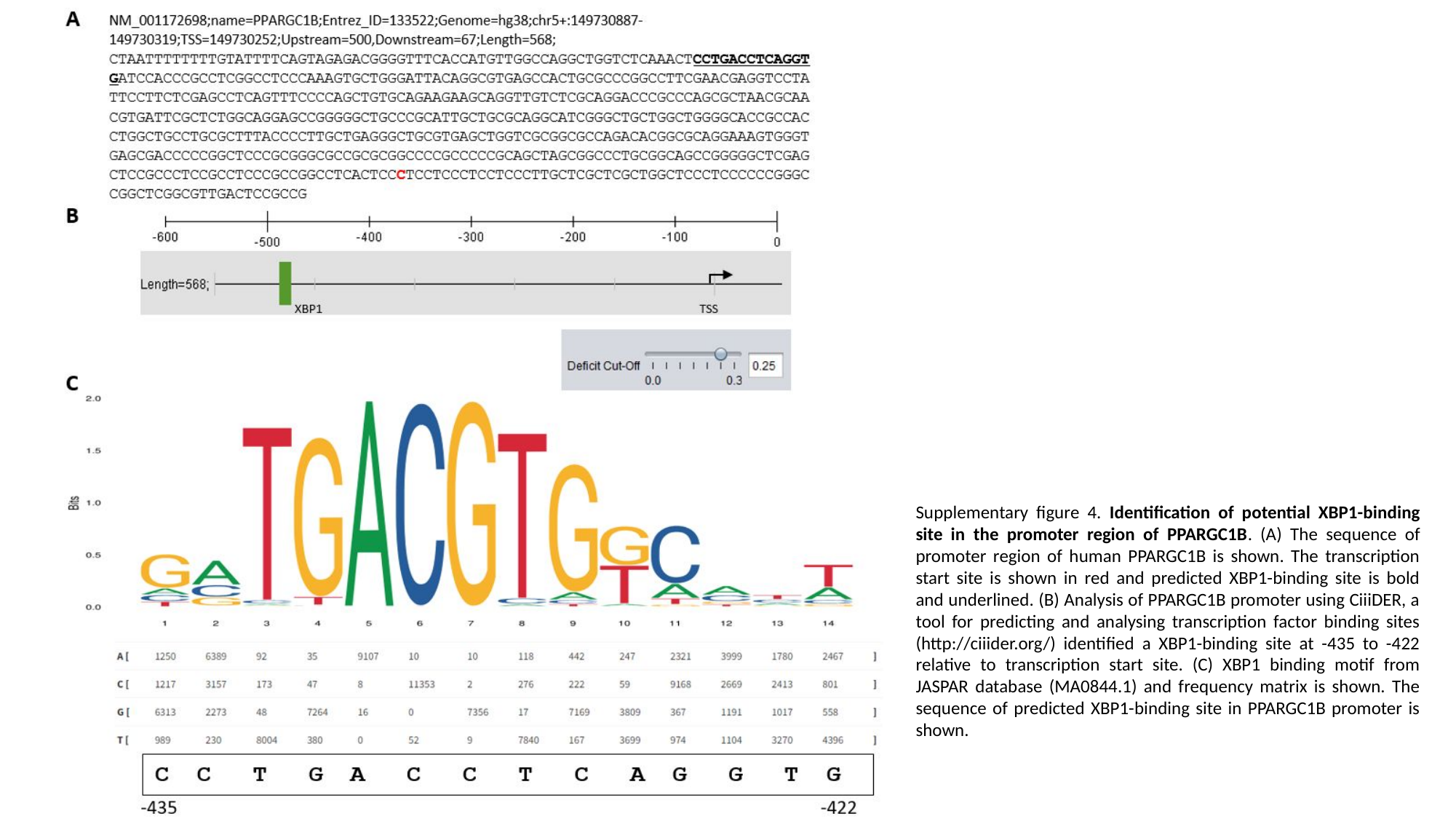

Supplementary figure 4. Identification of potential XBP1-binding site in the promoter region of PPARGC1B. (A) The sequence of promoter region of human PPARGC1B is shown. The transcription start site is shown in red and predicted XBP1-binding site is bold and underlined. (B) Analysis of PPARGC1B promoter using CiiiDER, a tool for predicting and analysing transcription factor binding sites (http://ciiider.org/) identified a XBP1-binding site at -435 to -422 relative to transcription start site. (C) XBP1 binding motif from JASPAR database (MA0844.1) and frequency matrix is shown. The sequence of predicted XBP1-binding site in PPARGC1B promoter is shown.

## Slide 5
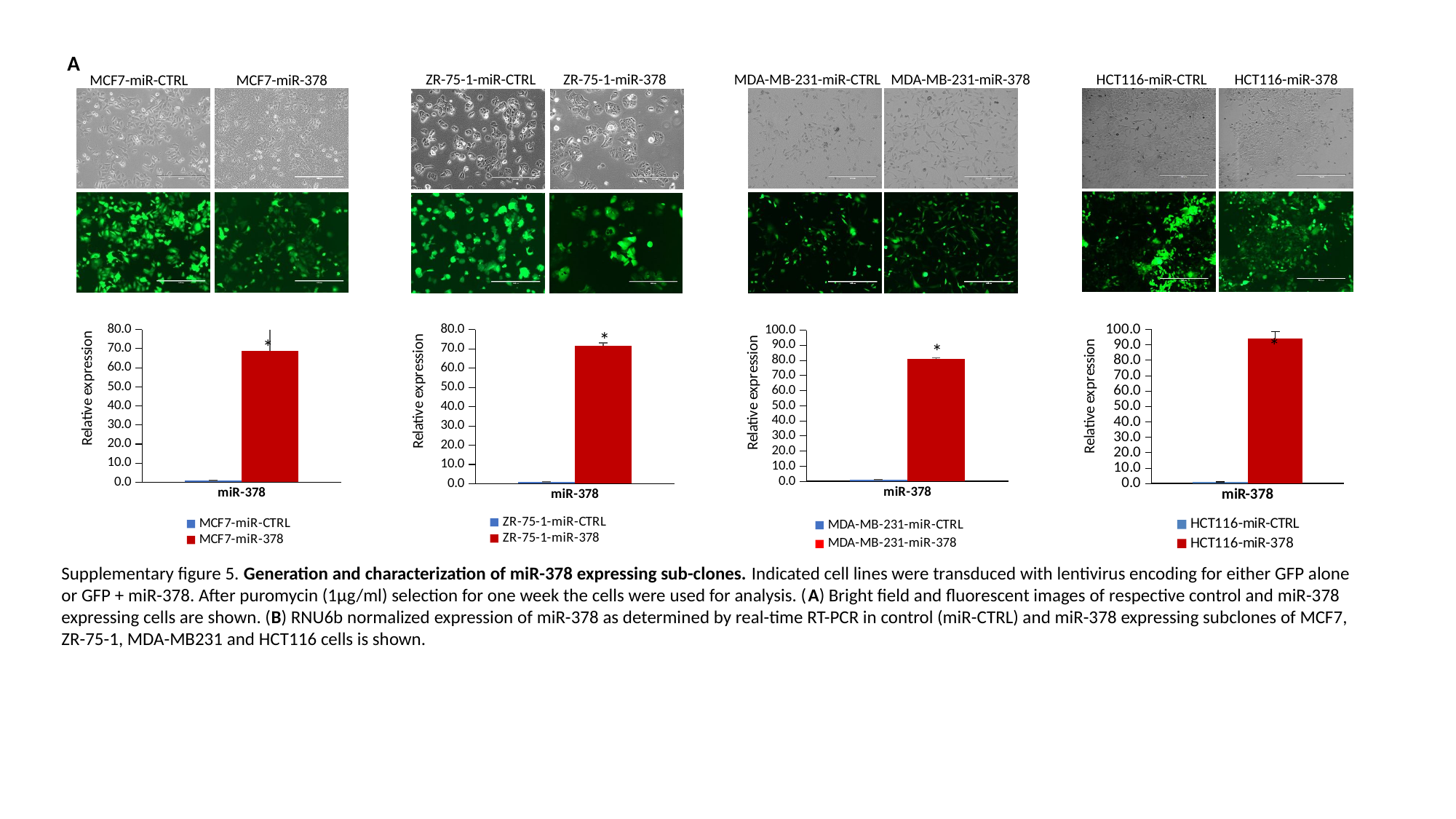

A
B
MDA-MB-231-miR-CTRL MDA-MB-231-miR-378
 HCT116-miR-CTRL HCT116-miR-378
 ZR-75-1-miR-CTRL ZR-75-1-miR-378
MCF7-miR-CTRL MCF7-miR-378
### Chart
| Category | MDA-MB-231-miR-CTRL | MDA-MB-231-miR-378 |
|---|---|---|
| miR-378 | 0.9998652456101359 | 80.78511409736929 |
### Chart
| Category | MCF7-miR-CTRL | MCF7-miR-378 |
|---|---|---|
| miR-378 | 1.0006201772609649 | 68.80117203564139 |
### Chart
| Category | ZR-75-1-miR-CTRL | ZR-75-1-miR-378 |
|---|---|---|
| miR-378 | 0.9996405714332018 | 71.69076267630739 |
### Chart
| Category | HCT116-miR-CTRL | HCT116-miR-378 |
|---|---|---|
| miR-378 | 1.0004340026413336 | 93.98121087181937 |*
*
Supplementary figure 5. Generation and characterization of miR-378 expressing sub-clones. Indicated cell lines were transduced with lentivirus encoding for either GFP alone or GFP + miR-378. After puromycin (1µg/ml) selection for one week the cells were used for analysis. (A) Bright field and fluorescent images of respective control and miR-378 expressing cells are shown. (B) RNU6b normalized expression of miR-378 as determined by real-time RT-PCR in control (miR-CTRL) and miR-378 expressing subclones of MCF7, ZR-75-1, MDA-MB231 and HCT116 cells is shown.

## Slide 6
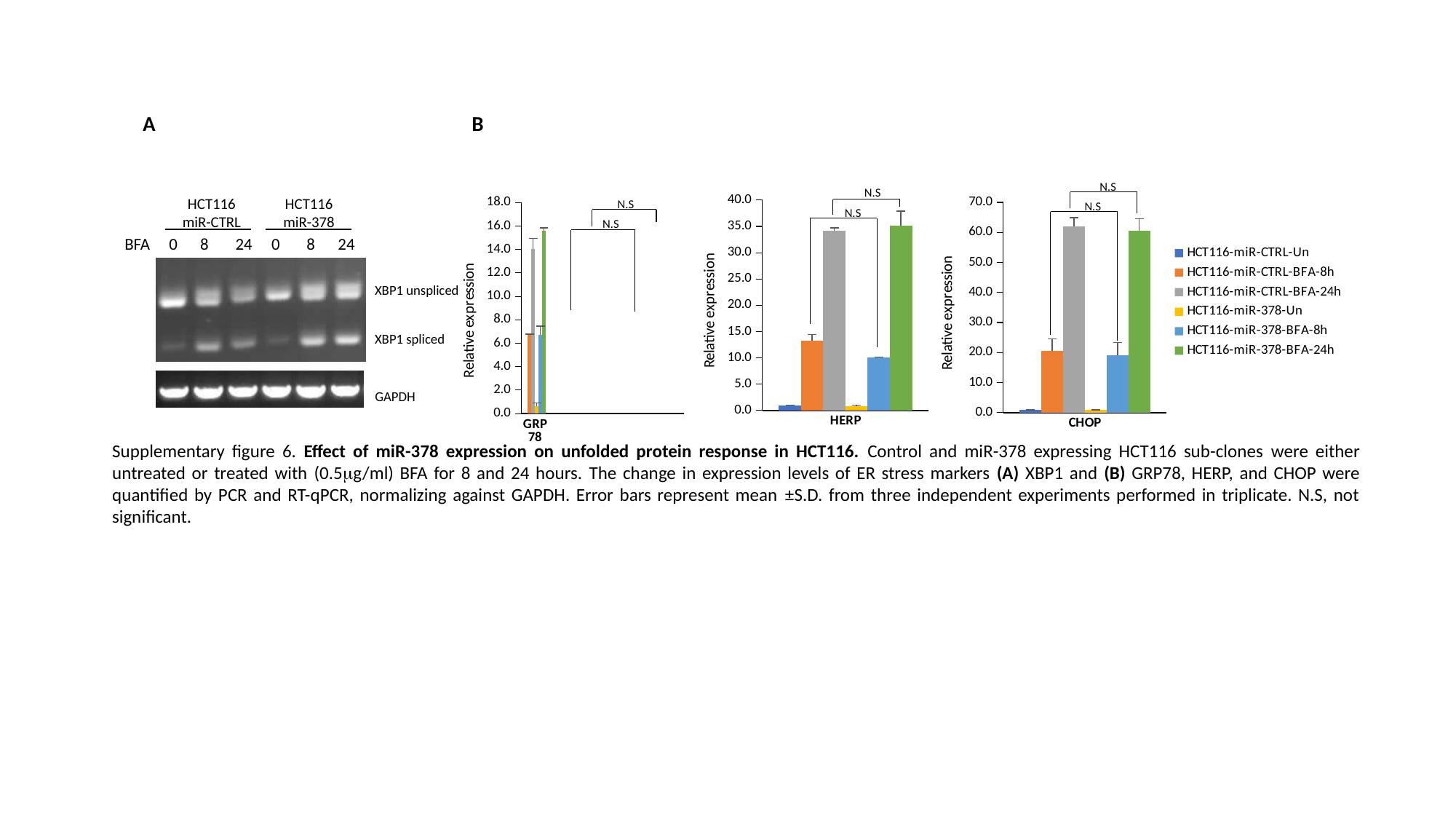

A B
N.S
### Chart
| Category | HCT116-miR-CTRL-Un | HCT116-miR-CTRL-BFA-8h | HCT116-miR-CTRL-BFA-24h | HCT116-miR-378-Un | HCT116-miR-378-BFA-8h | HCT116-miR-378-BFA-24h |
|---|---|---|---|---|---|---|
| CHOP | 0.9999957061175361 | 20.567932789868237 | 62.077374438918326 | 0.9540156774954673 | 19.220740759457886 | 60.65603380501091 |N.S
N.S
### Chart
| Category | HCT116-miR-CTRL-Un | HCT116-miR-CTRL-BFA-8h | HCT116-miR-CTRL-BFA-24h | HCT116-miR-378-Un | HCT116-miR-378-BFA-8h | HCT116-miR-378-BFA-24h |
|---|---|---|---|---|---|---|
| HERP | 1.0004263553280976 | 13.197294123990659 | 34.18863481090875 | 0.803597660501284 | 10.099485923910503 | 35.08572645832281 |N.S
### Chart
| Category | HCT116-miR-CTRL-Un | HCT116-miR-CTRL-BFA-8h | HCT116-miR-CTRL-BFA-24h | HCT116-miR-378-Un | HCT116-miR-378-BFA-8h | HCT116-miR-378-BFA-24h |
|---|---|---|---|---|---|---|
| GRP78 | 1.0002435663224807 | 6.69832486888755 | 14.023569511549779 | 0.6474750748463087 | 6.705771854263171 | 15.622982801069252 |N.S
N.S
HCT116
miR-378
HCT116
miR-CTRL
XBP1 unspliced
XBP1 spliced
GAPDH
BFA 0 8 24 0 8 24
Supplementary figure 6. Effect of miR-378 expression on unfolded protein response in HCT116. Control and miR-378 expressing HCT116 sub-clones were either untreated or treated with (0.5μg/ml) BFA for 8 and 24 hours. The change in expression levels of ER stress markers (A) XBP1 and (B) GRP78, HERP, and CHOP were quantified by PCR and RT-qPCR, normalizing against GAPDH. Error bars represent mean ±S.D. from three independent experiments performed in triplicate. N.S, not significant.

## Slide 7
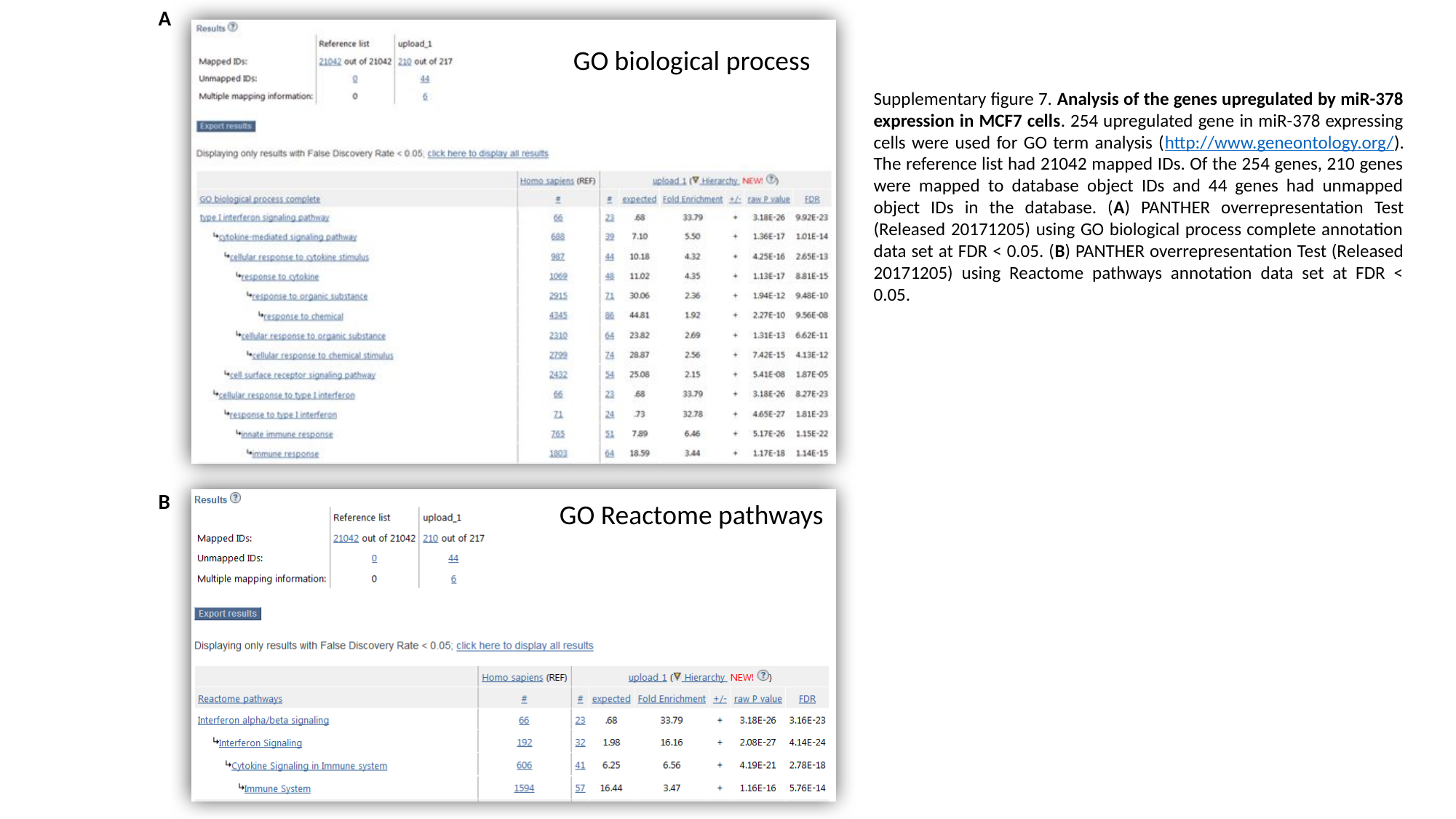

A
B
GO biological process
Supplementary figure 7. Analysis of the genes upregulated by miR-378 expression in MCF7 cells. 254 upregulated gene in miR-378 expressing cells were used for GO term analysis (http://www.geneontology.org/). The reference list had 21042 mapped IDs. Of the 254 genes, 210 genes were mapped to database object IDs and 44 genes had unmapped object IDs in the database. (A) PANTHER overrepresentation Test (Released 20171205) using GO biological process complete annotation data set at FDR < 0.05. (B) PANTHER overrepresentation Test (Released 20171205) using Reactome pathways annotation data set at FDR < 0.05.
GO Reactome pathways
